# Reporting preprints in the media during the COVID-19 pandemic

**DOI:** 10.1177/09636625221077392

**Published:** 2022-02-23

**Authors:** François van Schalkwyk, Jonathan Dudek

**Affiliations:** Stellenbosch University, South Africa; Leiden University, The Netherlands

**Keywords:** COVID-19, media representations, preprints, science journalism

## Abstract

Preprints have gained prominence in the dissemination of scientific findings. This development has been reinforced by the COVID-19 pandemic, which continues to require the rapid dissemination of new scientific information. However, since preprints usually have not undergone peer review, they lack the rigour of other scientific publications such as journal articles. This presents a challenge for the news media tasked with keeping the public informed about the latest scientific developments in the context of great uncertainty during a global pandemic. This research note investigates the reporting of scientific information from preprints in 80 news articles identified in news articles related to COVID-19 published in four South African online media outlets. Our results show that despite the publication of guidelines for reporting on preprints in the media, there is still a way to go regarding the judicious use of scientific information from preprints by the news media.

## 1. Introduction

The COVID-19 pandemic has had a profound impact on the communication of science (Grant, 2021). Scientists who are eager and perhaps impatient – sometimes legitimately so – for their findings to be published post-haste and for scientific information to be shared, are increasingly sharing their findings in preprints (Sever et al., 2019; Vale, 2015). As a result, preprints have taken on an important role in the rapid dissemination of science (Chiarelli et al., 2019) and during the COVID-19 pandemic in particular (Coates, 2021; Fraser et al., 2021; Sohrabi et al., 2021).

The uncertainty surrounding COVID-19 has fuelled a torrent of communication activity in the media as claims and speculations are published and debated. In this context, the provisional science of preprints provides another source of uncertainty (Coates, 2021). Adding to both the volume and levels of uncertainty are the perverse incentives of the attention economy (Myllylahti, 2020; Nixon, 2020; Tufekci, 2013) driving the spread of misinformation (Carlson and Harris, 2020; Price, 2018; Tufekci, 2013). This creates a challenge for the news media in particular.

On one hand, the news media is expected to report objectively to provide the public with factually accurate information (International Federation of Journalists (IFJ), 2019). On the other hand, journalists are under pressure from multiple quarters to report more rapidly, more frequently and in ways that attract attention. In such a communication landscape, the question is to what extent the reporting of scientific information extracted from preprints is qualified.

Previous studies have covered this topic by taking into account how preprints were reported in the Brazilian media (Oliviera et al., 2021), and, more generally, in the anglophone media (Fleerackers et al., 2021). Expanding our knowledge of how preprints serve as a source of information in the news media, our study focuses on the situation in South Africa and asks the following question: How have preprint articles related to COVID-19 been reported in the South African news media?

## 2. Literature review

Preprints are those scientific publications made available online prior to their formal publication (e.g. as journal articles or book chapters). In some cases, preprints are post-peer-reviewed articles accepted for publication and published online prior to the publication of the final version of record. In most cases, however, preprints are articles that have not undergone peer review and are self-published ‘informally’ by authors. Preprint servers provide the online infrastructure to host preprints and allow for the early registration of scholarly outputs to support collaborative and networked-based scientific endeavours, and to increase the speed of access and discovery.

According to Xie et al. (2021), since the launch of the arXiv preprint server in 1991, the number of preprints has increased rapidly, although preprints account for only 4% of research articles. Preprints are published on average 14 months earlier than research articles; and 41% of preprints are eventually published as a peer-reviewed journal article.

What is unprecedented during the COVID-19 pandemic is the number of preprint articles published (Fraser et al., 2021; Kousha and Thelwell, 2020). This is perhaps unsurprising given the intense demands placed on science as policymakers and the public seek scientific information. In this context, the availability of preprint articles has played an important role in advancing scientific discovery *and* in providing the latest scientific information about the virus to politicians and the public (Horbach, 2020).

Several cases of inaccurate research related to COVID-19 published in preprints have been reported (Heimstädt, 2020; Marcus and Oransky, 2020). A recent study found that much of the discussion (and even policymaking) about COVID-19’s transmissibility was driven by preprints rather than peer-reviewed literature (Majumder and Mandl, 2020). Many of the risks relate to the extent to which preprints are a trustworthy source of scientific information, particularly for non-experts (Coates, 2021). The fact that guidelines have been published for journalists on how to report on preprints (Avissar-Whiting, 2020; Hanage and Lipsitch, 2020; Helmuth, 2020; Ordway, 2020a, 2020b; Sheehan and Funk, 2020) attests to an awareness of the potential risks of open access to uncertified science (Osman et al., 2018). Recommendations that appear frequently in these guidelines are that journalists should indicate the provisional nature of findings reported in preprints and should solicit and quote the opinions of independent experts (see Table 1 in the Supplemental material).

Empirical studies show that the news media has not yet adapted sufficiently. Fleerackers et al. (2021) studied how COVID-19-related scientific information from preprints was communicated by 15 international digital content providers in developed anglophone countries. They found the contextualisation of information to be broadly lacking. Nevertheless, out of the 100 news stories featuring COVID-19-related preprints analysed, approximately half the stories emphasised uncertainty. Oliviera et al. (2021) found that 38.6% of a set of articles from the Brazilian media featured attempts to state that the findings still had to undergo scientific evaluation, while 27.6% failed to do so. This leads us to ask the following research questions:

1. Are preprint articles being used in the South African news media to report on the COVID-19 pandemic?
2. Is the provisional nature of the findings published in preprint articles indicated in the news media?
3. Are additional views from other sources provided in the news media when reporting on preprint articles?

## 3. Method

### Creating a set of news articles that mention preprints

Given the growing importance of online news in South Africa (Newman, 2019), four online news websites were selected for analysis: Health24, TimesLive (TL), Independent Online (IOL) and The Daily Maverick (DM).

In order to familiarise the research team with journalistic practice with regard to preprints, we first searched for news articles mentioning preprints in data from Altmetric. Altmetric is a service that, among other metrics, identifies news articles that mention scientific studies. Querying news mentions of COVID-19-related publications collected by Altmetric and filtering for the news sources in question and the period defined (7 January to 6 July 2020) resulted in 18 accessible news articles mentioning preprints. From those, it became apparent that news articles often did not use the term ‘preprint’ when referring to the source of the scientific information. This made it clear that it would not be possible to rely on queries using only the term ‘preprint’ to identify relevant news articles.

We created a second set of online news articles using the Pear Africa media monitoring service. From the Pear Africa data, 22,707 articles were extracted from the same media outlets that contained one or more of the following keywords: corona*, covid*, lockdown. The corpus of articles was again limited to the period 7 January to 6 July 2020. A second filter was then applied to identify only those news reports that referred to scientific articles. This was done by running a search query using the terms ‘study’, ‘studies’, ‘research’, ‘preprint’, ‘pre-print’, ‘paper’, ‘publication’ and ‘report’. All articles where the author was indicated as ‘afp’, ‘AFP’, ‘AFP Relaxnews’, ‘Reuters’ or ‘Trending’ were removed to include only news articles authored by South African journalists or contributors. Locally syndicated news articles were included as were articles that were likely to have been subjected to ‘strong editorial filters’ (Jaklevic, 2020). This process returned a total of 3227 news articles (IOL: 330, DM: 1470, TL: 847, Health24: 580).

Aware that we could not rely solely on filtering the news articles using the term ‘preprint’, the 3227 news articles were analysed manually for mentions of journal and preprint articles. After duplicates and incomplete records were removed, we were left with 2684 articles. This was followed by a close reading of each article. This analysis resulted in 426 articles that referenced COVID-19 science (e.g. journal articles) and 80 that mentioned preprints. Out of 2684 articles, only news articles that mentioned a preprint were used in the subsequent content analysis, that is, a total of 80 news articles which represents all South African authored news articles mentioning preprints in the four media outlets for the period 7 January to 6 July 2020. For the purposes of identifying preprints, a preprint article was defined as any article published online, and which reports findings following a scientific method and according to accepted conventions in terms of how such findings are presented, and which had not been peer reviewed at the time of its publication.

### Content analysis

A code book was developed deductively, drawing on published guidelines related to the reporting on preprints (see Table 1 in the Supplemental material). Two code groups were identified: (1) provisionality (i.e. the extent to which journalists indicate that the findings reported in preprints are uncertified and therefore provisional) and (2) multiple sources (i.e. the extent to which journalists provide additional information from other expert sources in relation to the preprint findings). The codes for provisionality were as follows: (1) clear statement of provisionality (SP4), (2) suggestion of provisionality (SP3), (3) ‘preprint’ or ‘not peer reviewed’ without explanation (SP2), (4) no provisionality (SP0), and (5) misunderstanding of preprint or preprint server (SP1). Sources were coded by type: scientists, scientific article or journal, politician, organisation, medical professional, media, citizens and nonmedical professionals and not specified.

The codes were tested by two coders who independently coded the 18 news articles in the Altmetric set. Cohen’s kappa was used to measure intercoder reliability because there were no missing values and no indication of great variance for any single set of codes. Cohen’s kappa was calculated in IBM SPSS Statistics 27 to measure intercoder reliability for the two code groups: (1) provisionality and (2) multiple sources. For provisionality, there was almost perfect agreement between the two coders with κ = .906; for multiple sources, the agreement was moderate (κ = .423; Viera and Garrett, 2005).

The news articles for coding were converted from web pages to PDF format and imported into the software package Atlas.ti Version 9 (Windows). Two coders independently coded all the news articles in the set. The coders compared their results manually, discussed any variation, and came to an agreement on the final coding for each article. This resulted in a single master set which was used to analyse the results in Atlas.ti and in MS Excel.

## 4. Findings

Out of the 2684 news articles that mentioned either a journal article or a preprint, only 2.98% (80) articles mentioned a preprint, in a total of 114 unique preprints. Table 1 shows that in a minority of the articles, there was some form of provisionality provided as a signal to readers to treat the findings with caution: in 5% of cases, provisionality was clearly stated, while in 11% it was merely suggested that the findings from the preprint should be taken as provisional. In 24% of news articles, authors used the term ‘preprint’ or stated that the scientific article referred to had not been peer reviewed, but provided no explanation to the readers as to what a preprint is or what the implication of describing a scientific article as a preprint is. In 59% of news articles, no statement of provisionality was provided, and findings were often attributed to ‘a study’ or ‘report’.

**Table 1.** Statements of provisionality in relation to preprints on COVID-19 reported in the South African online news media.

Statement type	No.	Percent	Examples
Clear statement of provisionality (SP4)	6	5	‘How reliable is this information? The team mentions that their study is a preprint and has not been peer reviewed. This means it has yet to be evaluated and should therefore not be used as clinical guidance’.
Suggestion of provisionality (SP3)	12	11	‘suggested that’, ‘preliminary findings’
‘Preprint’ or ‘not peer reviewed’ without explanation (SP2)	27	24	‘The results were published on a preprint server called ChemRxiv’, ‘a preprint study’, ‘not yet peer reviewed’
Misunderstanding of preprint or preprint server (SP1)	2	2	‘The new study was published online on the open-data site SSRN’
No provisionality (SP0)	67	59	‘study’, ‘report’
Total	114	101^a^	

a Greater than 100% due to rounding.

In the 80 news articles analysed, 233 other related sources were mentioned. Table 2 shows that of the sources referred to 57% as scientific, that is, either a scientist quoted (30%) or a reference to a scientific publication (27%). In 13% of cases, news articles cited comments from an organisation such as the World Health Organization (WHO). In 12% of cases, news articles provided less specific information about their sources, referring to them as ‘experts’, ‘researchers’, ‘studies’ or other nonspecific descriptors. Citizens or nonmedical professionals (5%), politicians or governments (4%) and medical professionals (3%) were infrequent sources quoted.

**Table 2.** Sources cited on the topic of COVID-19 in the South African news media.

Source type	No. of mentions	% of mentions
Scientist	69	30
Scientific article or journal	63	27
Organisation (e.g. WHO)	31	13
Not specified (e.g. experts, researchers, a study)	27	12
Media	15	6
Citizens or nonmedical professional	12	5
Politician or government	9	4
Medical professional (e.g. doctor)	7	3
Total	233	100

WHO: World Health Organization.

## 5. Discussion

Despite the exponential increase in the number of COVID-19-related preprints published (Fraser et al., 2021; Torres-Salinas et al., 2021), our analysis in the South African context showed that the news media relies on preprints only to a limited degree. This may suggest a heavy reliance on science news syndicated from the overseas media – content which was excluded from our analysis – given the reported decline in science desks in South Africa (Van Zuydam, 2018). It may also confirm that despite the increase, the relative share of preprints in the larger corpus of scientific papers remains small (Brainard, 2021). On one hand, this finding could be taken to indicate that the extent of the public’s exposure to uncertified science related to the COVID-19 pandemic is relatively low. Consequently, the risk of uncertified science being misinterpreted and widely disseminated may also remain low. All the more so when the communities of attention for preprints on social media platforms comprise mostly academics (Carlson and Harris, 2020). On the other hand, it is not only the quantum of news reports that determines whether a news item attracts readers’ attention; other factors such as highly selective and frequent posting of content may amplify the attention it receives (Van Schalkwyk, 2019).

We found that 59% of online news articles did not provide a statement of provisionality when reporting on a preprint. This proportion is higher than found to be the case by Oliviera et al. (2021) – 27% of news in the Brazilian media – and by Fleerackers et al. (2021: Table 4) – 42.5% of news articles provided no indication of provisionality. Our finding that 24% of news articles either used the term ‘preprint’ or stated that the scientific article referred to had not been peer reviewed, but provided no explanation to readers as to what a preprint is or what the implications of non-review are, are consistent with those of Fleerackers et al. (2021: Table 5). This value is, however, much lower than the 52% reported by Oliviera et al. (2021).

In a minority of cases, a clear statement of provisionality was provided. While limits on news article word counts may preclude overly lengthy explanations of what preprints are and why they should be treated with caution, it would be possible to make use of the affordances of online reporting to provide hyperlinks to more detailed explanations published elsewhere.

Taken together, our findings suggest that the South African news media does not appear to be following emerging guidelines specifically related to the reporting of scientific information from preprints. The reasons behind the deviance from professional norms require further investigation.

## 6. Limitations

Two limitations should be noted. First, the analysis relied on a relatively small number of news media articles mainly due to geographic filtering and the limited period of 6 months. Second, the 6-month timeframe was deemed to be too short to explore any possible change over time in journalistic practice in relation to the reporting of preprints. It is possible that reporting practice will change over time as journalists (and scientists) become more familiar with preprints as a new type of scientific publication, including the benefits and risks of relying on preprints for scientific information.

## 7. Conclusion

During the first 6 months of the COVID-19 pandemic, South African media relied on science published in preprints, but to a limited degree and not necessarily in place of peer-reviewed journal articles. In general, the provisional nature of the scientific findings published in preprints was not signalled to readers. Other experts were, however, quoted in relation to the findings reported in preprints.

Preprints are likely to remain a feature in the formal science communication system. The COVID-19 pandemic and the concomitant need for constant updates on the effects and treatment of the virus, as well as the urgent need for rapid advancement in scientific knowledge, has made available to the media a new source of scientific information.

An increase in scientific publications, which are yet to undergo scrutiny by peers and are therefore provisional in terms of their claims, raises the risk that such claims are repeated in the news in undifferentiated or uncritical ways. The risk may extend to use by ideologically motivated groups. This calls for caution and for the judicious use of science without compromising the benefits of its openness. Paramount in this is the responsible reporting of scientific information from preprints in the news media.

## Supplemental Material

sj-docx-1-pus-10.1177_09636625221077392 – Supplemental material for Reporting preprints in the media during the COVID-19 pandemic Click here for additional data file. Supplemental material, sj-docx-1-pus-10.1177_09636625221077392 for Reporting preprints in the media during the COVID-19 pandemic by François van Schalkwyk and Jonathan Dudek in Public Understanding of Science
