# Supplementary material for: Reporting preprints in the media during the COVID-19 pandemic
Source: Public Underst Sci. 2022 Feb 23;31(5):608–16. doi: 10.1177/09636625221077392 (PMC9160779; doi:10.1177/09636625221077392)
Supplement: sj-docx-1-pus-10.1177_09636625221077392 – Supplemental material for Reporting preprints in the media during the COVID-19 pandemic [file sj-docx-1-pus-10.1177_09636625221077392.docx]

**Reporting preprints in the media during the Covid-19 pandemic: Supplemental material**

François van Schalkwyk, South African Research Chair in Science Communication, Centre for Research on Evaluation, Science and Technology (CREST), Stellenbosch University, Private Bag X1 Matieland, Stellenbosch 7602, South Africa | [fbvschalkwyk@sun.ac.za](mailto:fbvschalkwyk@sun.ac.za) | <http://orcid.org/0000-0002-1048-0429>

Jonathan Dudek, Centre for Science and Technology Studies (CWTS), Leiden University, Wassenaarseweg 62A, 2333 AL Leiden, The Netherlands | <http://orcid.org/0000-0003-2031-4616>

**Table A: Codes derived from the literature on journalistic best practice**

| **Recommendation** | **Source** | **Code group** | **Codes** |
| --- | --- | --- | --- |
| Journalists should avoid characterizing preprint findings as established facts.  make it clear that preprint findings are preliminary. | Ordway (2020a) | **Provisionality (SP)**: Qualification of the scientific information extracted from the preprint by stating the provisional nature of the findings | 1. No provisionality 2. Suggestion of provisionality 3. Mentions ‘preprint’ or ‘not peer reviewed’ without explanation 4. Clear statement of provisionality 5. Misunderstanding of preprint or preprint server |
| Where appropriate, help readers understand how the healthcare system works, how science works, how scientific publishing works, how the immune system works, how viruses work. | Helmuth (2020) |  |  |
| Research has shown that a lack of labeling can lead to reader confusion … sophisticated news consumers, especially when reading online, have difficulty discerning taxonomic distinctions between “report”, “investigation”, “op-ed” and “opinion” | Iannucci & Adair (2017) |  |  |
| Readers might not heed caveats about “early” or “preliminary” evidence, Woloshin said. “The problem is, once it gets out into the public it’s dangerous because people will assume it’s true or reliable, and I don’t think that’s true.” Woloshin suggested news organizations refrain from saying a preprint was “published,” which wrongly signals that a manuscript “must have gone through some sort of editorial review.” | Jaklevic (2020) |  |  |
| it would help even more to have solicited and quoted the opinion of at least one independent expert and include any caveats they may have. a  make a habit of conferring with researchers.  Call other scientists in the same field and ask if they rate the work in the preprint as credible …  a journalist should have a group of experts she can go to for advice, while remembering that even experts aren’t experts in everything. | Ordway (2020a) | **Other sources (MS)**:  Multiple sources of information to confirm, refute, contextualise or question the findings presented | 1. Scientist 2. Scientific article or journal 3. Organisation 4. Not specified 5. Media 6. Citizen or non-medical professional 7. Politician or government 8. Medical professional |
| take time to track down researchers with knowledge and experience in the topic they’re covering. When reporting on new health topics such as the Covid-19, which experts worldwide are scrambling to understand, it’s a good idea to interview multiple researchers. | Ordway (2020b) |  |  |
| asking other researchers to comment on the quality of the methodology of the paper can be very informative.  give readers a sense of what other papers in the same field have found and whether those conclusions are in line with the preprint findings. | Khamsi (2020) |  |  |
| it’s still up to journalists to do that scrutiny, including reaching out to independent experts and checking to see whether other research concurs. | Jaklevic (2020) |  |  |
| So that means that as journalists sift through the deluge of preliminary information, they have to take a critical eye to these early reports. Question the researcher, talk to other scientists | Hamilton (2020) |  |  |
| Seek diverse sources of information. Because no one has digested everything about the state of the epidemic, different experts will know different things and see different holes in our reasoning. | Hanage & Lipsitch (2020) |  |  |
| journalists find out whether its authors are reputable and have previously done high-quality research | Ordway (2020a, 2020b) | Preprint author information | 1. Name(s) of preprint author(s) 2. Institutional affiliation of preprint author(s) |

**References**

Haelle T (2020) Tips on covering preprints about coronavirus research. [Blog post]. *Association of Health Care Journalists*. <https://healthjournalism.org/resources-tips-details.php?id=1113#.Xu_FTy2ZPxU>.

Hamilton E (2020) How should journalists cover coronavirus preprint studies? University of Wisconsin-Madison [website]. <https://news.wisc.edu/how-should-journalists-cover-coronavirus-preprint-studies/>.

Hanage B and Lipsitch M (2020, February 23) How to report on the Covid-19 outbreak responsibly. *Scientific American*. <https://blogs.scientificamerican.com/observations/how-to-report-on-the-covid-19-outbreak-responsibly/>.

Iannucci, R., & Adair, B. (2017, August 15). Reporters’ Lab study finds poor labeling on news sites. *Duke Reporters’ Lab*. <https://reporterslab.org/news-labeling-study-results-media-literacy/>

Jaklevic MC (2020, April 1) Strong caveats are lacking as news stories trumpet preliminary Covid-19 research. [Blog post]. *Health News Review*. <https://www.healthnewsreview.org/2020/04/strong-caveats-are-lacking-as-news-stories-trumpet-preliminary-covid-19-research/>.

Khamsi R (2020) What best practices are you following in covering preprints during the pandemic? [Blog post]. *Health Journalism*. [https://healthjournalism.org/core-topic.php?id=10andpage=sharedwisdom](https://healthjournalism.org/core-topic.php?id=10&page=sharedwisdom).

Ordway D-M (2020a, April 2) Covering biomedical research preprints amid the coronavirus: 6 things to know. *Journalist’s Resource*. <https://journalistsresource.org/tip-sheets/research/medical-research-preprints-coronavirus/>.

Ordway D-M (2020b, March 6) Covering Covid-19 and the coronavirus: 5 tips from a Harvard epidemiology professor. *Journalist’s Resource*. <https://journalistsresource.org/studies/society/public-health/covid-19-coronavirus-epidemiology/>.
